# Crucial parameters for precise copy number variation detection in formalin‐fixed paraffin‐embedded solid cancer samples

**DOI:** 10.1002/1878-0261.70192

**Published:** 2025-12-23

**Authors:** Hanne Goris, Vasiliki Siozopoulou, Léon C van Kempen, Anne Sieben, Ella Roelant, Stig Hellemans, Elyne Backx, Laure Sorber, Koen De Winne, Senada Koljenović, Karen Zwaenepoel

**Affiliations:** ^1^ Department of Pathology Antwerp University Hospital Belgium; ^2^ Center for Oncological Research (CORE), Molecular Imaging, Pathology, Radiotherapy & Oncology (MIPRO) University of Antwerp Belgium; ^3^ Department of Pathology Ziekenhuis aan de Stroom Antwerp Belgium; ^4^ Department of Pathology, Maastricht University Maastricht University Medical Center The Netherlands; ^5^ Clinical Trial Center (CTC) CRC Antwerp, Antwerp University Hospital University of Antwerp Belgium; ^6^ Department of Computer Science, Adrem Data Lab University of Antwerp Belgium

**Keywords:** cancer diagnostics, copy number variations, FFPE tissue, SNP array, ULP‐WGS

## Abstract

Copy number variations (CNVs) play a crucial role in cancer diagnostics and prognostics, potentially impacting treatment decisions. Ultra‐low‐pass whole‐genome sequencing (ULP‐WGS) has emerged as a promising alternative to array‐based methods for CNV detection, especially in formalin‐fixed paraffin‐embedded (FFPE) samples. However, sequencing biases and sample heterogeneity necessitate the optimization of CNV detection tools for FFPE sample‐derived data. This study evaluates three open‐source CNV callers (CNVpytor, ichorCNA, and WisecondorX) using ULP‐WGS and compares their performance against a single nucleotide polymorphism (SNP) array. Our results demonstrate that under optimal experimental conditions, ichorCNA and WisecondorX achieved equal detection of true positive results, with reduced false positive results compared to the SNP array. The SNP array detection pattern differed somewhat from that of the CNV callers, while ichorCNA and WisecondorX had the most comparable detection pattern. We highlight the importance of (pre‐)analytical parameters such as neoplastic cell content, sequencing coverage, and bin size selection on CNV detection accuracy. Our findings support the adoption of ULP‐WGS‐based CNV detection as a robust alternative to SNP arrays, with WisecondorX emerging as the most suitable tool for clinical implementation.

## Graphical abstract

This study shows that copy number variations (CNVs) can be reliably detected in formalin‐fixed paraffin‐embedded (FFPE) solid cancer samples using ultra‐low‐pass whole‐genome sequencing, provided that key (pre)‐analytical parameters are optimized. Two CNV callers matched or exceeded the performance of a standard array method, demonstrating the potential for utilizing FFPE samples in genomic studies and cancer diagnostics.

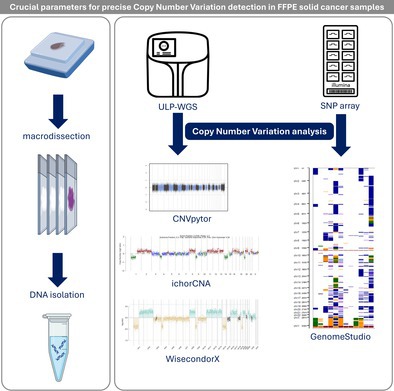

**Abbreviations**
absCN: absolute copy number
BAF: B‐allele frequency
cfDNA: cell‐free DNA
CN: copy number
CNV: copy number variation
FFPE: formalin‐fixed paraffin‐embedded
FP: false positive
gDNA: genomic DNA
Het_DEL: heterozygous deletion
HLAMP: high‐level amplification
Hom_DEL: homozygous deletion
LB: liquid biopsy
LLAMP: low‐level amplification
LOD: limit of detection
MAD: mean absolute deviation
PON: panel of normals
QC: quality control
SNP: single nucleotide polymorphism
TAT: turnaround time
TP: true positive
ULP‐WGS: ultra‐low‐pass whole‐genome sequencing

## 1 Introduction

Copy number variations (CNVs) are genomic structural variations involving the duplication or deletion of DNA segments, leading to differences in the number of copies of these regions between cells. CNVs can occur in both germline (inherited) and somatic (acquired) contexts, with somatic CNVs being particularly prevalent in cancer genomes, where amplifications of oncogenes (copy number gain) or deletions of tumor suppressor genes (copy number loss) can drive malignancy. CNV analysis is relevant in current molecular diagnostics for a growing number of indications [1, 2]. The detection of CNVs can (i) aid in diagnosis and tumor (sub)typing (e.g., *MDM2* amplification in liposarcoma [3], 1p19q codeletion in oligodendroglioma [4, 5, 6, 7]), (ii) guide treatment decisions (e.g., *ERRB2* amplification in metastatic breast cancer [8, 9, 10, 11], *MET* amplification in lung cancer [12, 13, 14]), (iii) have prognostic value (e.g., *CDKN2A* homozygous deletion as an independent predictor of poor prognosis in glioma [15, 16, 17]) and (iv) facilitate the development of novel therapeutic strategies tailored to the individual genomic landscape of each patient's tumor [18, 19].

Formalin‐fixed, paraffin‐embedded (FFPE) samples are extensively used in molecular diagnostics and research due to their ability to preserve tissue morphology over long periods. However, current CNV analysis methods and bioinformatics tools are not tailored to FFPE samples. These methods do not account for guanine–cytosine (GC) content bias, the presence of fixation artifacts, and sample heterogeneity. Validation of sample, sequencing, and bioinformatics tools for CNV calling in FFPE samples is therefore required. Array‐based CNV identification, either from array‐based comparative hybridization or SNP array approaches, is valuable for detecting CNVs, but faces several limitations when applied to FFPE samples, such as a large amount of high‐quality DNA and introduction of hybridization noise. These shortcomings can impact the accuracy and reliability of CNV detection. Due to the reducing costs associated with sequencing technologies, ultra‐low‐pass whole‐genome sequencing (ULP‐WGS) is rapidly evolving into the preferred methodology for discerning CNVs (> 10 kb) [20, 21]. The technique offers a comprehensive approach for genome‐wide screening of CNVs, with potential applications in large‐scale population studies, clinical research, and personalized oncology.

CNV calling algorithms can be based on one or more approaches: read pair, read depth, split read, or assembly algorithms [22]. Most CNV calling tools are based on read depth algorithms [23], which predict CNVs by comparing the read depth of a sample against a reference to identify significant deviations across the genome. Calling CNVs can be challenging due to the amount of input DNA for library preparations, the ratio of normal cells and neoplastic cells in the sample, tumor heterogeneity, and sequencing coverage. Among frequently used CNV callers that use a depth of coverage‐based analytical approach to predict CNVs from ULP‐WGS are CNVpytor, ichorCNA, and WisecondorX. CNVpytor is a software package for detecting CNVs based on depth of coverage and allele imbalance in whole‐genome sequencing data [24, 25]. When coverage depth is sufficient (30×), the accuracy and reliability of CNV detection can be enhanced by analysis of SNP and indel data. CNVpytor is not dependent on a panel of normals (PON) and can be used for a range of input matrices including cell‐free DNA (cfDNA) isolated from liquid biopsies (LB) and genomic DNA (gDNA) isolated from FFPE tissue or fresh‐frozen tumor (FFT) tissues. The software package ichorCNA estimates the fraction of tumor in cell‐free DNA and predicts large‐scale CNVs from ULP‐WGS [26, 27]. ichorCNA can be used for LB, FFPE tissue, and FFT tissues. ichorCNA can be run without a PON. Implementation of a PON may reduce noise and improve accuracy. Using samples for a PON that were processed and sequenced similarly may correct for systematic biases arising from library construction, sequencing platform, and FFPE‐specific artifacts [28]. WisecondorX is an evolved version of WISECONDOR, a software package for detecting CNVs in ULP‐WGS data [29, 30]. Like CNVpytor and ichorCNA, WisecondorX can be used for cfDNA, gDNA, LB, FFPE tissue, and fresh‐frozen tissues. A PON is necessary for analysis. WisecondorX has shown to be performant on FFPE samples for CNV detection, but a validation of generated results in a large cohort is not yet demonstrated.

The aim of this study was to optimize and validate a CNV detection method in FFPE tumor samples using ULP‐WGS. We optimized the abovementioned open‐source CNV callers for ULP‐WGS and compared their CNV calling performance to a SNP array. The impact of pre‐analytic and analytic parameters (e.g., age of sample, neoplastic cell content, DNA input quantity, and bin size) was assessed. The best performing CNV caller was selected and used for validation.

## 2 Material and methods

### 2.1 Sample selection and preparation

To set up a PON for ichorCNA and WisecondorX analysis, 32 non‐tumor skin samples were used. The training dataset contained ten tumor samples with 26 known CNVs (Table S1) and ten non‐tumor skin samples [31]. The ten tumor samples consisted of nine FFPE tumor samples and one reference FFPE tumor sample (Seraseq Solid Tumor CNV Mix, +3 copies, SeraCare, LGC Clinical Diagnostics). The independent validation dataset consisted of 19 tumor samples with 37 known CNVs (Table S1). All known CNVs were previously identified with clinically validated techniques (i.e., immunohistochemistry, fluorescence *in situ* hybridization, targeted next‐generation sequencing, and SNP array analysis).

To assess the robustness of CN profiling, 128 FFPE samples of different tissue types were selected. Quality control (QC) was assessed using the mean absolute deviation (MAD) metric, calculated with ichorCNA. A MAD threshold of < 0.15 was chosen based on literature [27, 32] to indicate acceptable quality, with higher values reflecting high data noise. To determine the impact of neoplastic cell content and the CNV limit of detection (LOD), 41 melanoma samples (24 primary and 17 metastatic tumors) and two glioma samples were used, respectively. To determine the LOD, a twofold serial dilution series was prepared in triplicate by using a wild‐type FFPE sample that was brought to the same concentration as the glioma samples.

DNA was isolated from FFPE tissue blocks using macrodissection. From each block, a hematoxylin–eosin‐stained section (5 μm thickness) and five unstained sections (10 μm thickness) were prepared. The region of interest was marked using the Philips Intellisite Pathology Solution (v3.2). The percentage of neoplastic cells was estimated by a pathologist as part of routine practice, with an experienced pathologist (VS) providing an independent assessment. In cases of discrepancy, a third pathologist (AS) was consulted for resolution. The indicated region of interest was manually dissected from blank slides. gDNA was isolated using the QIAcube and QIAmp DNA FFPE Tissue Kit (QIAGEN, Hilden, Germany) according to manufacturer's instructions. The gDNA concentration was measured using the Qubit dsDNA BR Assay Kit with a Qubit 3.0 Fluorometer (Invitrogen, Thermo Fisher Scientific, Waltham, MA, USA).

This study was approved on 22/08/2022 (reference ID 2022‐3699) by the UZA‐UAntwerp Ethics Committee. The study was conducted in concordance with the Declaration of Helsinki and ICH Good Clinical Practice. The FFPE samples were originally obtained between August 2018 and July 2025, and all samples were retrieved from the UZA archive. As this study was non‐interventional and samples were collected retrospectively, the requirement for written informed consent was waived in accordance with applicable regulations [33, 34, 35].

### 2.2 SNP array

Degraded FFPE gDNA (40–200 ng) was restored with the Infinium HD FFPE Restore Kit (Illumina, San Diego, CA, USA) and the DNA Clean & Concentrator Kit (Zymo Research, Irvine, CA, USA) according to the manufacturer's instructions. The Infinium HD Assay Ultra was used to produce amplified and fragmented gDNA able to hybridize to the HumanCytoSNP‐12 v2.1 BeadChip (Illumina). After single‐base extension of the oligos on the BeadChip, the BeadChip was stained and scanned with the Illumina iScan system. The SNP array data was visualized and analyzed with the GenomeStudio (v2.0.5, Illumina) with a bin size of 500 kb, confidence threshold = 35, minimum probe count = 3, and HumanCytoSNP‐12 v2.1 cluster and manifest files. In addition to applying an acceptable call rate of 95%, more stringent settings for optimal FFPE sample analysis included filtering on confidence ≥ 200, minimum probe count = 10, and blacklisting the region Chr9: 40 294 324–42 374 011.

### 2.3 ULP‐WGS

gDNA was fragmented using the NEBNext dsDNA Fragmentase (New England BioLabs, Ipswich, MA, USA) to produce fragments of around 150 bp. A NGS library was prepared using the Ion Xpress™ Plus Fragment Library Kit and Ion Xpress™ Barcode Adapters (Thermo Fisher Scientific, Waltham, MA, USA) according to manufacturer's instructions. The NGS library was equalized at 100 pM and loaded onto a 540 chip with the Ion Chef System (Thermo Fisher Scientific) to achieve a minimal total sequencing coverage of 10 million bp per sample. ULP‐WGS was performed with the Ion GeneStudio S5 Prime sequencer (Thermo Fisher Scientific) using 500 flows.

### 2.4 Bioinformatic analysis of ULP‐WGS data

Bioinformatic analysis was performed with the Torrent Suite software (v5.16.1, Thermo Fisher Scientific) [36]. Sequence reads were mapped against the hg19 reference genome using the Torrent Mapping Alignment Program with default settings [37]. The generated BAM files were further analyzed with CNVpytor (v1.2.1), ichorCNA (v0.2.0, QC30 mapping) and WisecondorX (v1.2.5) to generate CN profiles and CNVs for each sample. An example of the generated CN profiles of one sample is presented in Fig. S1A–C. A PON was created for ichorCNA and WisecondorX using 32 non‐tumor skin samples. All CNVs in the sex chromosomes were removed and adjacent genomic aberrations with similar CNV type were merged. The coverage depth did not allow us to perform CNV calling with CNVpytor from B‐allele frequency (BAF) data. Samtools (v1.13) was used to artificially reduce the total coverage depth of a sample. The coverage depth of the original BAM file was reduced to 50%, 25%, and 10% in triplicate using three different seeds.

### 2.5 Statistical analysis and data visualization

All statistical tests and data visualization were done with the NumPy, pandas, scikit‐learn, Matplotlib, and Seaborn libraries in Python (v3.10), using Jupyter Notebook (v6.5.5). Normality was checked graphically and with the Shapiro–Wilk test. When comparing two groups, the Welch's *t*‐test was used in case of normally distributed data (as assumptions for equal variances were not met) and the Mann–Whitney *U*‐test in case of skewed or small data sets. When testing for significant differences between more than two groups, a Kruskal–Wallis test was performed (since assumptions for normality were not met), followed by a post hoc Dunn's test with Bonferroni correction. A *P*‐value < 0.05 was considered significant. Hierarchical clustering was performed to compare the CNV detection patterns of the SNP array and CNV callers with each other. The clustering was done with the linkage function from the SciPy library using the Ward method, which aims to minimize the total within‐cluster variance. The performance was evaluated by calculating the detection rate.

## 3 Results

### 3.1 Performance of ULP‐WGS to detect CNVs in FFPE samples

ULP‐WGS was performed on a training dataset of 20 FFPE samples, including nine tumor samples and one reference sample, as well as ten non‐tumor skin samples (Table S1). The total number of mapped reads for these samples ranged from 7 to 16 million, corresponding to sequencing depths of 0.3× to 0.8×. Among the nine tumor samples, 14 CNVs were previously identified using clinically validated techniques (i.e., immunohistochemistry, fluorescence *in situ* hybridization, targeted next‐generation sequencing, and SNP array analysis). The reference sample contained 12 CNVs. Overall, these FFPE samples included 26 known CNVs (true positives, TP): 16 low‐level amplifications (LLAMP), four high‐level amplifications (HLAMP), three homozygous deletions (Hom_DEL), and three heterozygous deletions (Het_DEL) (Table 1).

**Table 1:** CNV detection with SNP array and different CNV callers applying different confidence thresholds. Number of true positives in nine tumor samples and one reference sample, and false positives in one reference sample and ten non‐tumor samples is given. CNV detection was performed with SNP array and three CNV callers (CNVpytor, ichorCNA, and WisecondorX), using the default and more stringent filter settings with a bin size of 500 kb. AMP, amplification; DEL, deletion; Het_DEL, heterozygous deletion; HLAMP, high‐level amplification; Hom_DEL, homozygous deletion; LLAMP, low‐level amplification.

CNV type	Default settings				Filter settings			
	SNP array	CNVpytor	ichorCNA	WisecondorX	SNP array	CNVpytor	ichorCNA	WisecondorX
True Positives (*N* = 26)	12 (46%)	10 (38%)	17 (65%)	12 (46%)	12 (46%)	9 (35%)	12 (46%)	11 (42%)
LLAMP (*N* = 16)	5 (31%)	3 (19%)	9 (56%)	4 (25%)	5 (31%)	3 (19%)	4 (25%)	3 (19%)
HLAMP (*N* = 4)	4 (100%)	1 (25%)	2 (50%)	2 (50%)	4 (100%)	0 (0%)	2 (50%)	2 (50%)
Hom_DEL (*N* = 3)	2 (67%)	3 (100%)	3 (100%)	3 (100%)	2 (67%)	3 (100%)	3 (100%)	3 (100%)
Het_DEL (*N* = 3)	1 (33%)	3 (100%)	3 (100%)	3 (100%)	1 (33%)	3 (100%)	3 (100%)	3 (100%)
False Positives	36	27	32	1	9	17	1	0
Off‐target AMP	31	0	16	1	9	0	1	0
Off‐target DEL	5	27	16	0	0	17	0	0

CN profiles were generated using CNVpytor, ichorCNA, and WisecondorX, with a bin size of 500 kb. Using the default settings, CNVpytor, ichorCNA, and WisecondorX detected 10 (38%), 17 (65%), and 12 (46%) true positives (TP), respectively (Table 1). Each CNV caller successfully detected all six deletions. However, they varied in identifying the 20 amplifications (AMP), with CNVpytor detecting four AMP (20%), ichorCNA detecting 11 AMP (55%), and WisecondorX detecting six AMP (30%). Additionally, the default settings yielded 27 false positives (FP) for CNVpytor, 32 FP for ichorCNA, and one FP for WisecondorX.

**Fig. 1:**
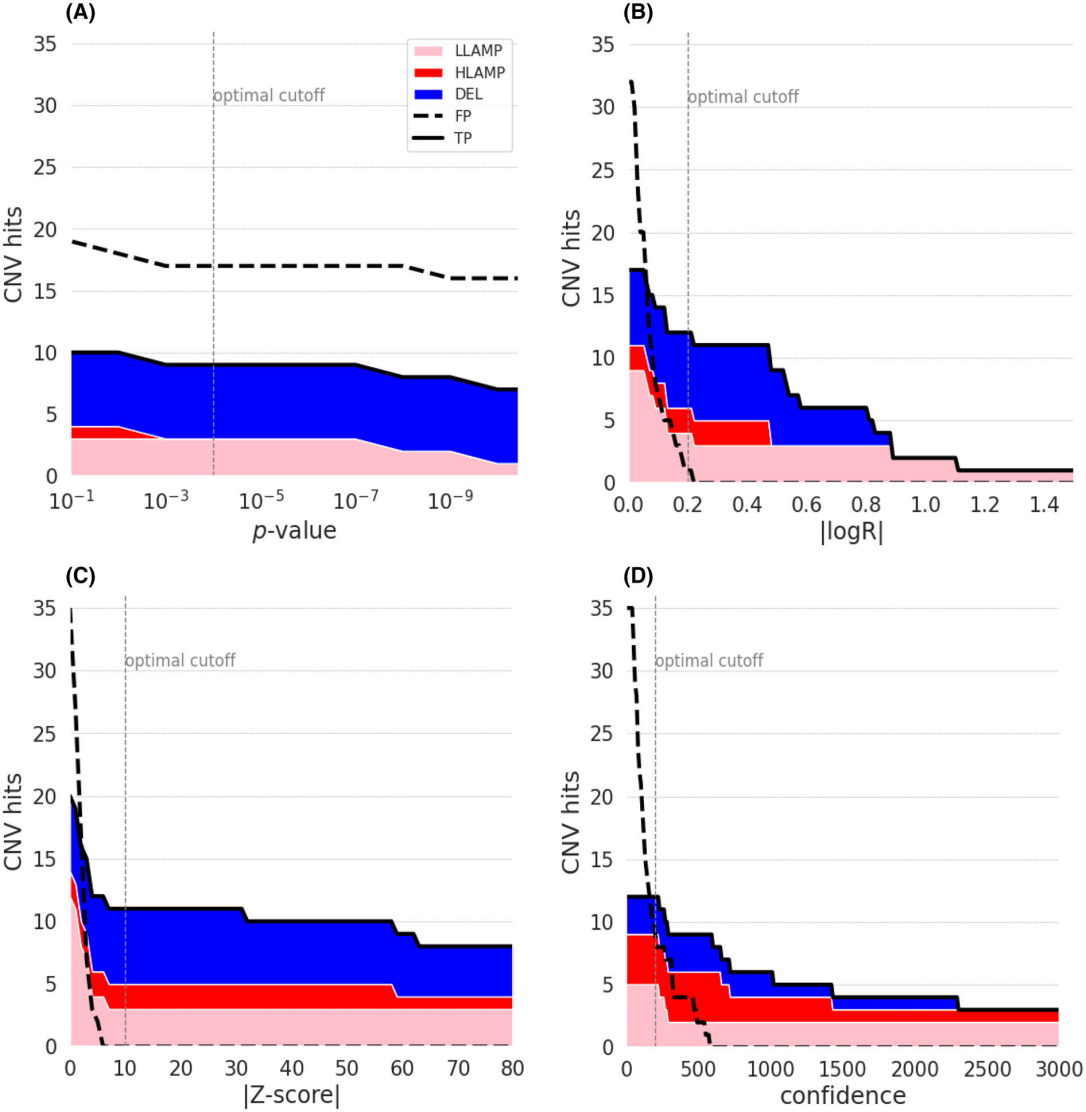
Performance of SNP array versus ULP‐WGS to detect CNV in FFPE samples. Distribution of true positive (TP, solid black line) and false positive (FP, dashed black line) copy number variations (CNVs) detected across various confidence scores. The TP consist of LLAMP (pink), HLAMP (red), and DEL (blue). The vertical dashed line marks the used confidence threshold (default values: CNVpytor = 10^−4^, ichorCNA = 0.2, WisecondorX = 5, SNP array = 35). (A) CNVpytor, (B) ichorCNA, (C) WisecondorX, (D) SNP array results. AMP, amplification; DEL, deletion; Het_DEL, heterozygous deletion; HLAMP, high‐level amplification; Hom_DEL, homozygous deletion; LLAMP, low‐level amplification.

To determine the optimal filter settings for FFPE samples, we applied varying values for the respective confidence threshold parameters of the CNV callers (*P*‐value for CNVpytor, logR for ichorCNA, and *Z*‐score for WisecondorX) and analyzed the number of TP and FP (Fig. 1, Panels A–C) and the average number of CNV for the non‐tumor samples. The ideal detection threshold was established at the point where the TP were maximized while ensuring that the average CNV for non‐tumor samples remained below one. For CNVpytor, the used confidence threshold was *P*‐value = 0.0001, detecting nine TP (zero HLAMP, three LLAMP, six DEL) and 17 FP in the reference FFPE sample. For ichorCNA, |logR| = 0.2 was used as confidence threshold, detecting 12 TP (two HLAMP, four LLAMP, six DEL) and one FP in the reference FFPE sample. For WisecondorX, the used filter setting was |*Z*‐score| = 10, detecting 11 TP (two HLAMP, three LLAMP, six DEL) without any FP in the reference FFPE sample (Table 1).

### 3.2 Performance of SNP array versus ULP‐WGS to detect CNV in FFPE samples

The ability to detect 26 TP CNVs was also evaluated by SNP array (hymancytoSNP‐12 v2.1, Illumina). With default settings (bin size 500 kb, confidence threshold = 35, minimum probe count = 3), the SNP array detected 12 TP (five LLAMP, four HLAMP, three DEL). Additionally, 36 FP were identified in the reference FFPE sample (31 AMP, five DEL). By applying more stringent settings (confidence threshold = 200, minimum probe count = 10) to mitigate these off‐target effects, we achieved a reduction to nine FP, while maintaining the detection of all 12 TP (see Table 1, Fig. 1D).

**Fig. 2:**
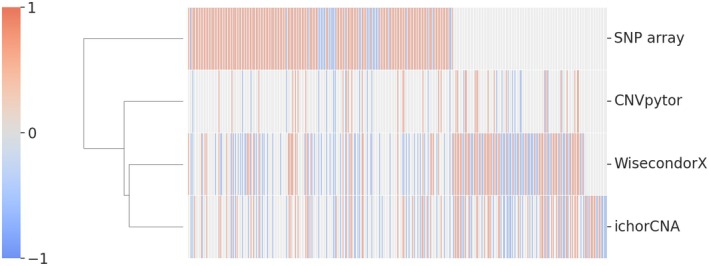
CNV detection pattern of CNV callers. Heatmap visualization of hierarchical clustering of CNV detection in ten FFPE samples across four CNV callers: ichorCNA, WisecondorX, CNVpytor, and SNP array. Each row represents a genomic region, with different colors indicating amplifications (red/1), neutral (white/0), or deletions (blue/−1). The SNP array column is dominated by red lines, indicating a higher number of amplifications in contrast to ichorCNA, WisecondorX, and CNVpytor that show a more balanced pattern.

A comparative analysis of the CNV output from four CNV callers applied to ten FFPE samples of the training set revealed notable differences in their CNV detection patterns. The SNP array method detected a high number of AMP (*N* = 257) and fewer DEL (*N* = 48). WisecondorX and ichorCNA detected fewer AMP (*N* = 142 and 110, respectively) but more DEL (*N* = 144 and 122, respectively); CNVpytor exhibited a distinct pattern, detecting only 48 AMP and 32 DEL (Fig. 2). Overall, the hierarchical clustering suggests that WisecondorX and ichorCNA have the most similar CNV detection profiles, while CNVpytor stands out for detecting the fewest CNVs and SNP array for detecting the most CNVs, particularly AMP.

### 3.3 Impact of pre‐analytical parameters of FFPE samples on ULP‐WGS and CN profiling

To assess the robustness of CN profiling, ULP‐WGS analysis of 128 FFPE tissue samples was conducted. The evaluated pre‐analytical parameters were specimen type, FFPE tissue block age, gDNA input, and total coverage depth. CN profiles with a MAD less than 0.15 were considered to be of good quality (= successful). Among the 128 samples, 110 were biopsies (85.9%) and 18 were resection specimens (14.1%). The FFPE tissue block age was up to 5 years (Suppl. Table S2), except for one sample that was 8 years old. The median age of the biopsies was 2 years, while the median age of the resections was zero years. The gDNA assay input varied from 6.4 to 336 ng (median = 97.9 ng). Coverage depths ranged from 0.001× to 1.7× (median = 0.47×), and MAD scores ranged from 0.04 to 0.68 (median = 0.08). Only seven (5.5%) samples (five biopsies and two resections, including the only sample of 8 years old) exceeded the MAD cutoff of 0.15, indicating that FFPE samples can provide sufficient reads with low noise.

**Fig. 3:**
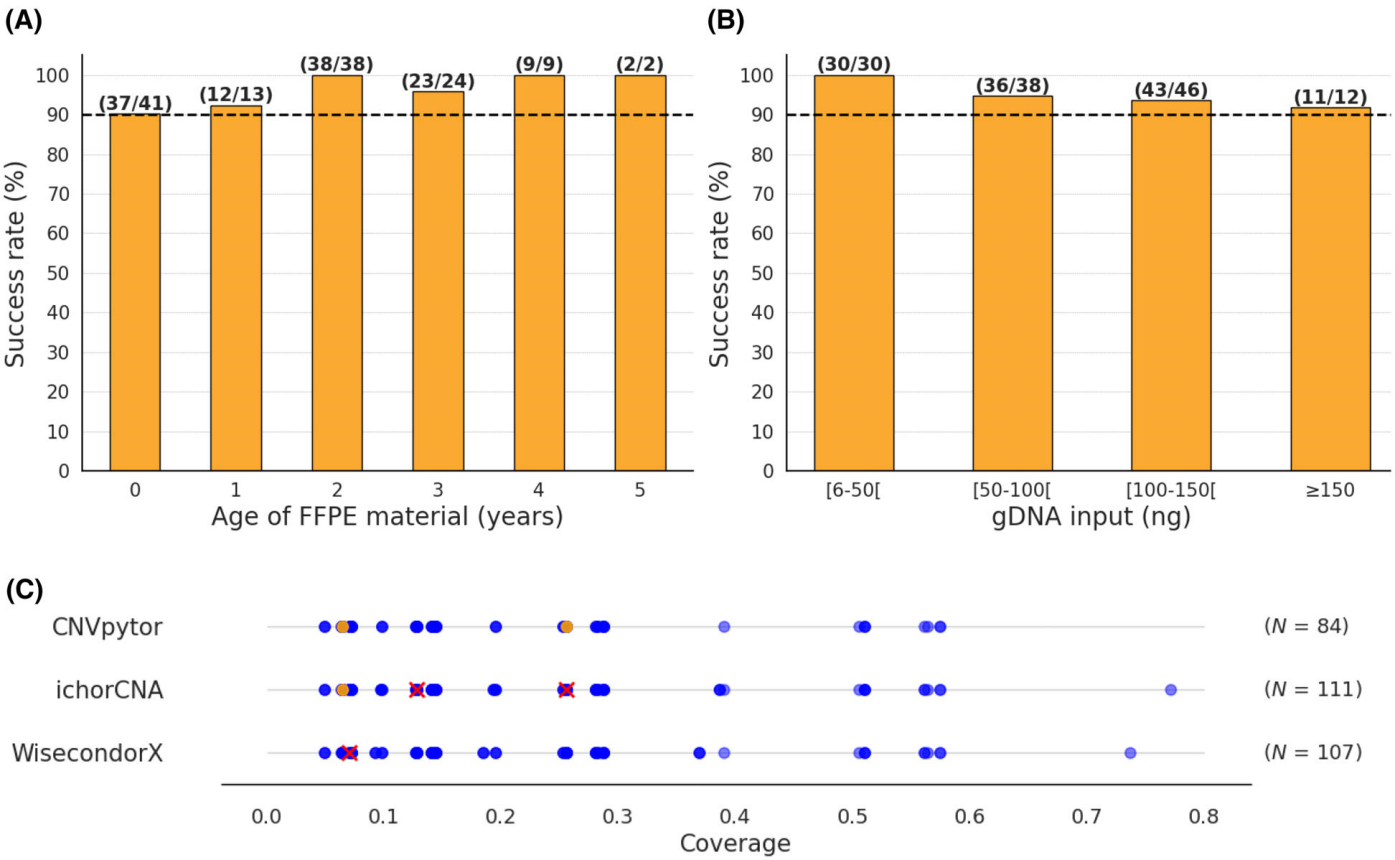
Impact of pre‐analytical parameters on CN profiling. (A) Effect of FFPE tissue block age (years) and (B) gDNA input quantity (ng) on success rate (%). The number of successful samples over the total number of samples within the respective group is displayed above each bar. A 90% success rate threshold is indicated by the black horizontal line, showing that success rate remains above this benchmark for all sample ages tested, including materials up to 5 years old and below 50 ng of gDNA input. (C) Scatter plot illustrating the detection of CNVs across varying coverage for three CNV callers: CNVpytor, ichorCNA, and WisecondorX. The sequencing coverage of ten samples was artificially reduced in triplicate. The *X*‐axis represents the absolute coverage (x), while the *Y*‐axis categorizes the CNV callers. Blue dots (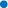) indicate CNVs that were detected in all three different seeds, orange dots (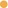) indicate CNVs that were detected in one or two different seeds, while red “x” markers (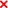) represent CNV that were detected in none of the three seeds. The coverage was artificially reduced in triplicate using samtools (v1.13). CNVs that were not detected at the highest, thus not diluted, coverage were not included in the figure. *N*, number of included data points for each CNV caller.

The success rate was defined as the number of samples that passed QC, divided by the total number of samples. When stratifying the samples by specimen type (biopsies and resections), age of the FPPE tissue block (one to 5 years), and gDNA input (in steps of 50 ng), a success rate of > 90% was consistently observed across all groups (Fig. 3, Panels A to B). These findings underscore the robustness of the assay, demonstrating that both biopsy and resection specimens are suitable for analysis, even when the FFPE tissue block is up to 5 years old. The optimal gDNA input was determined to be 50 ng, although successful analysis was possible with inputs as low as 6 ng. Samples that did not pass QC exhibited a 10‐fold lower coverage depth (median = 0.05×) compared to those that did (median = 0.48×), reinforcing the validity of our findings.

To evaluate the impact of the total coverage depth on CNV detection, the sequencing coverage of nine tumor FFPE samples and one reference FFPE tumor sample from the training dataset were artificially reduced in triplicate. The coverage depth was systematically halved (100–50–25–12.5%), resulting in a coverage depth ranging from 0.771× to 0.035×. All samples were analyzed using the three CNV callers, employing the filter settings established in section *3.1*. CNVs that were not detected at 100% coverage depth were not taken into further analysis. Notably, only WisecondorX successfully detected all TPs in all three replicates above 0.1× coverage, while ichorCNA and CNVpytor required a minimal coverage of 0.25× (Fig. 3C). The reduction in total coverage had minimal impact on confidence scores. No new false positive CNVs appeared by reducing the coverage.

### 3.4 Impact of bin size on CNV detection

To determine the influence of bin sizes on CNV detection, samples of the training dataset were analyzed using various bin sizes (ranging from 100 kb to 2 Mb). For CNVpytor, adjusting the bin size from 250 kb to 1 Mb enhanced performance, as the detection of the TP remained stable while the number of FP decreased by more than half, with an average CNV of zero in the non‐tumor samples. In the case of ichorCNA, adjusting the bin size from 500 kb to 1 Mb resulted in the loss of one LLAMP and the gain of one HLAMP. The FP dropped from one to zero, while the average number of CNVs in non‐tumor samples remained one, indicating that both 500 kb and 1 Mb are effective options, depending on whether sensitivity or specificity is prioritized in a diagnostic setting. For WisecondorX, increasing the bin size from 100 kb to 2 Mb led to the loss of three LLAMP and one HLAMP, with the FP rate reaching zero and the average number of CNV in the non‐tumor samples decreased from two to zero (see Table 2). A bin size of 250 kb resulted in the best performance for WisecondorX, surpassing the other CNV callers by detecting the highest number of TP without generating any FP.

**Table 2:** Impact of bin size on CNV detection performance. Number of true positives in nine tumor samples and one reference sample, and false positives in one reference sample and ten non‐tumor samples across different bin sizes is given. CNV detection was performed with three CNV callers (CNVpytor, ichorCNA, and WisecondorX) using the filter settings determined in Performance of ULP‐WGS to detect CNVs in FFPE samples. AMP, amplification; DEL, deletion; Het_DEL, heterozygous deletion; HLAMP, high‐level amplification; Hom_DEL, homozygous deletion; LLAMP, low‐level amplification.

CNV type	CNVpytor			ichorCNA		WisecondorX				
	250 kb	500 kb	1 Mb	500 kb	1 Mb	100 kb	250 kb	500 kb	1 Mb	2 Mb
True Positives (*N* = 26)	9 (35%)	9 (35%)	9 (35%)	12 (46%)	12 (46%)	14 (54%)	14 (54%)	11 (42%)	11 (42%)	10 (38%)
LLAMP (*N* = 16)	3 (19%)	3 (19%)	3 (19%)	4 (25%)	3 (19%)	5 (31%)	5 (31%)	3 (19%)	3 (19%)	2 (13%)
HLAMP (*N* = 4)	0 (0%)	0 (0%)	0 (0%)	2 (50%)	3 (75%)	3 (75%)	3 (75%)	2 (50%)	2 (50%)	2 (50%)
Hom_DEL (*N* = 3)	3 (100%)	3 (100%)	3 (100%)	3 (100%)	3 (100%)	3 (100%)	3 (100%)	3 (100%)	2 (67%)	3 (100%)
Het_DEL (*N* = 3)	3 (100%)	3 (100%)	3 (100%)	3 (100%)	3 (100%)	3 (100%)	3 (100%)	3 (100%)	3 (100%)	3 (100%)
False Positives	22	20	9	1	0	1	0	0	0	0
Off‐target AMP	0	0	0	1	0	0	0	0	0	0
Off‐target DEL	22	20	9	0	0	1	0	0	0	0

### 3.5 Limit of detection for CNV detection

**Fig. 4:**
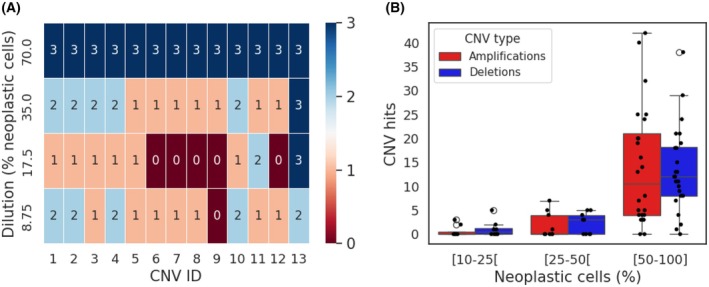
Effect of neoplastic cell content dilution on CNV detection. (A) Heatmap showing the detection of the 13 CNVs across varying dilution levels (70%, 35%, 17.5% and 8.75%). CNV ID 1–5 are LLAMP, CNV ID 6–12 are Het_DEL, and CNV ID 13 is a Hom_DEL. The numbers in the heatmap correspond with the amount of replicates detected (3 = detection in 3 replicates, 0 = no detection). (B) Distribution of amplifications (red boxes) and deletions (blue boxes) across different categories of neoplastic cell content (%). The 50–100% category differed significantly from the 10–25% category and 25–50% category (both *P* < 0.001, pairwise Mann–Whitney *U*‐test), indicating challenges in detecting CNVs at lower neoplastic cell content. There was no significant difference in CNV counts between the 10–25% category and 25–50% category (*P* = 0.25, pairwise Mann–Whitney *U*‐test), nor between amplifications and deletions (*P* = 0.32, Mann–Whitney *U*‐test). The boxplot shows the median values, the interquartile range (IQR), and the whiskers extending to the data points within 1.5 × IQR, with individual data points plotted as dots. ULP‐WGS data of 41 tumor samples was analyzed with the WisecondorX caller, using a 250 kb bin size and |*Z*‐score| = 10.

Based on the superior performance demonstrated by WisecondorX with a |*Z*‐score| of ten and a 250 kb bin size, we selected this CNV caller and filter settings for further validation. To determine the limit of detection (LOD), a serial dilution series was prepared in triplicate with two samples, containing 13 known CNVs (five LLAMP, seven Het_DEL, and one Hom_DEL). The selected samples had a high neoplastic cell content (70%) and DNA concentration (3.4 and 4.7 ng·μL^−1^) to ensure sufficient material for the dilution series. The LOD was defined as the lowest copy number (CN) at which the CNV can be detected in all three replicates. Except for the one Hom_DEL, a dilution from 70% to 35% resulted in the assay failing to reliably detect the LLAMP and Het_DEL in each replicate (Fig. 4A). More specifically, at 35% neoplastic cells, the majority of LLAMPs were detected in two of the three replicates, and the majority of Het_DEL were detected in one replicate. At 17.5% neoplastic cells, the majority of LLAMPs were detected in one of the three replicates, and the majority of Het_DEL were not detected. At 8.75% neoplastic cells, the majority of LLAMPs were detected in two of the three replicates, and the majority of Het_DEL were detected in one replicate. Only at 8.75% was the Hom_DEL not detected in all three replicates.

### 3.6 Impact of neoplastic cell content on CNV detection

The WisecondorX caller with a 250 kb bin size and a |*Z*‐score| of ten was applied on the ULP‐WGS data of 41 melanoma samples. The median number of CNVs in this sample set is 9. Samples were categorized based on neoplastic cell content into three categories: 10–25% (*N* = 8), 25–50% (*N* = 9), and 50–100% (*N* = 24). Of each category, the average CNV was calculated. The average number of detected CNV declined drastically when the neoplastic cell content was below 50%. Samples with less than 25% neoplastic cell content had an average of 1.8 CNV, those with 25–25% neoplastic cells had four CNVs and samples with 50–100% neoplastic cells had 28 CNVs. The 50–100% category differed significantly from the 10–25% category (*P* < 0.001) and 25–50% category (*P* < 0.001), indicating challenges in detecting CNVs at lower neoplastic cell content (Fig. 4B). There was no significant difference in CNV counts between the 10–25% category and 25–50% category (*P* = 0.25), nor between amplifications and deletions (*P* = 0.32).

### 3.7 CNV detection in an independent validation set

**Fig. 5:**
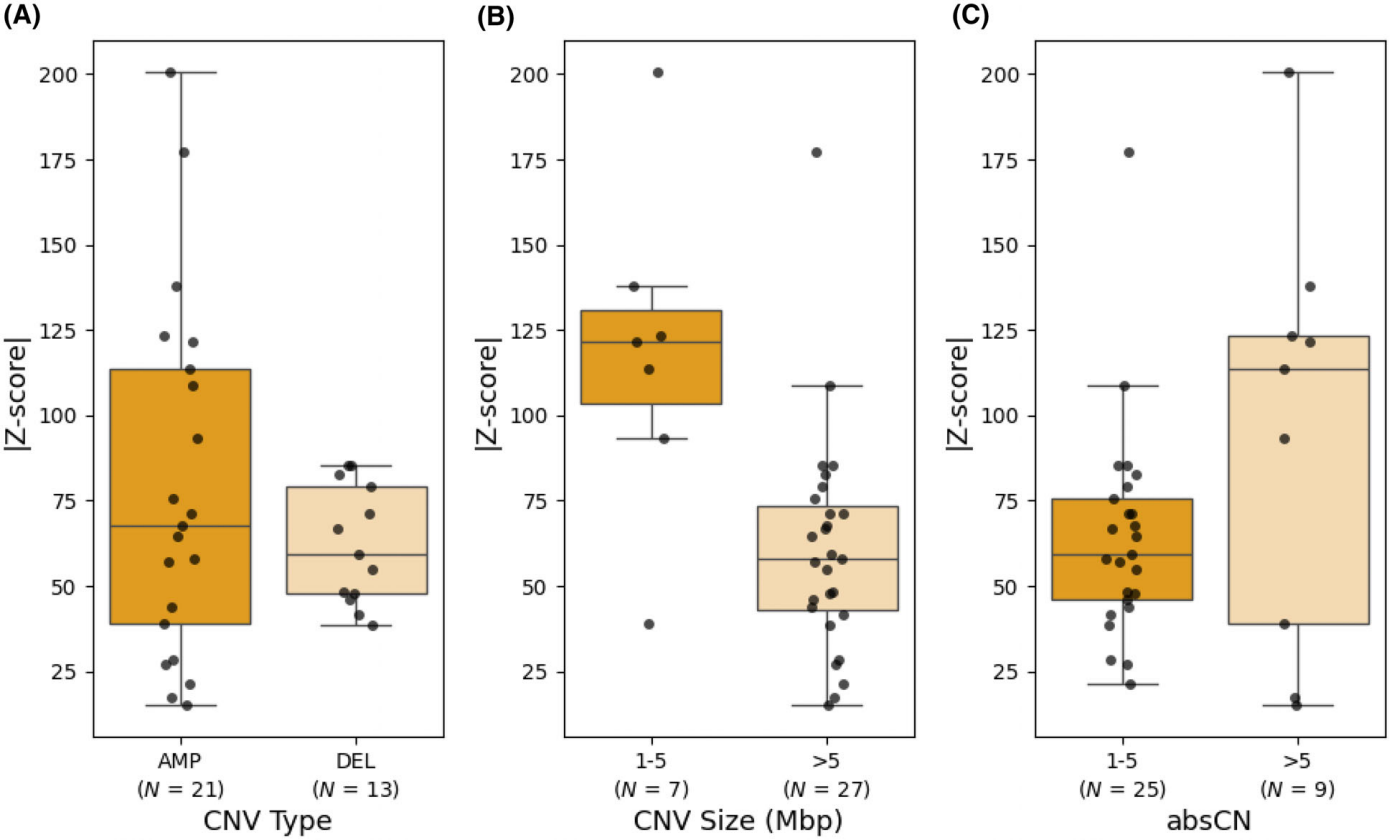
CNV detection in an independent validation set. Boxplots visualizing the distribution of (A) absolute *Z*‐scores across type of CNV, (B) CNV size, or (C) absolute copy number (absCN) of the 34 TP detected in the validation set. No significant difference was observed in the mean absolute *Z*‐score between deletions and amplifications (*P* = 0.178, Welch's *t*‐test), or between CNVs with a low and high absCN (*P* = 0.405, Mann–Whitney *U*‐test). A significant difference was observed between CNVs smaller and larger than five Mbp (*P* = 0.004, Mann–Whitney *U* test). The boxplots show the median values, the interquartile range (IQR), and the whiskers extending to the data points within 1.5 × IQR, with individual data points plotted as dots. A validation set of 19 FFPE tissue samples with 37 known CNVs was analyzed using WisecondorX with a 250 kb bin size and a |*Z*‐score| of 10. AMP, amplification; DEL, deletion.

A validation set of 19 FFPE tissue samples with 37 known CNVs was analyzed using WisecondorX with a 250 kb bin size and a |*Z*‐score| of 10. The neoplastic cell content of these samples varied between 20% and 90% (Suppl. Table S1). Of these, 34 CNVs (92%) were detected, including all deletions but missing three AMP (two LLAMP and one HLAMP) (Table 3). The three samples containing a missed AMP all had a neoplastic cell content of ≥ 50% and were less than 1 year old. The |*Z*‐score| of the detected CNVs, reflecting the confidence of the call, ranged from 15.0 to 200.5, with a mean of 72.6. Most CNVs exhibited a |*Z*‐score| well above the threshold, with only two CNVs having a |*Z*‐score| between ten and 20. No significant difference (*P* = 0.178) was observed between the mean |*Z*‐score| of deletions (62.0) and amplifications (79.1) (Fig. 5A). The size of the detected CNVs varied from 1 to 198 Mbp, reflecting both single genes and large genomic regions. The mean |*Z*‐score| for CNVs smaller than five Mbp was 118.5, while for CNVs larger than five Mbp, the mean |*Z*‐score| was 60.7 (Fig. 5B). A significant difference was observed in the distributions of the two groups (*P* = 0.004). The absolute copy number (absCN) of the detected CNV ranged from one to 30. The 26 CNVs with a low absCN (one to five) had a mean absolute *Z*‐score of 64.3, while those with high absCN (> 5) had a mean |*Z*‐score| of 95.7, indicating reliable detection even for CNVs with low CN changes (Fig. 5C). No significant difference was observed in the distributions of the two groups (*P* = 0.405).

**Table 3:** Detection of CNVs in an independent validation set. True positive rate (TPR) is given for CNV detection using WisecondorX with a bin size of 250 kb and a filter setting of |*Z*‐score| = 10. DEL, deletion; HLAMP, high‐level amplification; LLAMP, low‐level amplification.

CNV type	TPR	mean |*Z*‐score|	mean CNV size (Mbp)
True positives (*N* = 34)	31 (91%)	73.9	65.7
LLAMP (*N* = 17)	15 (88%)	82.4	73.8
HLAMP (*N* = 4)	3 (75%)	82.8	6.5
DEL (*N* = 13)	13 (100%)	62.0	69.9

## 4 Discussion

This study compared the performance of ULP‐WGS‐based CNV callers to a SNP array in FFPE samples. Our findings highlight the strong potential of ULP‐WGS‐based CNV callers as alternatives to SNP arrays, particularly with FFPE‐specific filter settings. By focusing on FFPE samples, the standard input for clinical diagnostic workflows, we provide insights directly relevant to routine practice. Comparative analyses with other tissue types, such as frozen samples, were not included in this study but remain a valuable topic for future work. Establishing a PON for ichorCNA and WiscondorX clearly adds value, as supported by literature on read depth analysis [38] and further reinforced by our own unpublished data. Among the ULP‐WGS‐based methods, WisecondorX detected the highest number of TP in the training dataset, while maintaining the lowest number of FP, making it the preferred option when accurate CNV detection with minimal FP is essential. All three ULP‐WGS‐based methods outperformed the SNP array in detecting deletions. The SNP array detected about 50% of TP events in the training dataset, with perfect detection for HLAMP but weaker performance for LLAMP and DEL. The relatively high number of FP (nine in total) suggests greater noise and misclassification of non‐CNV regions, underscoring the SNP array's tendency to generate incorrect CNV calls in FFPE samples. Compared to ichorCNA and WisecondorX, the SNP array detected a similar number of TP but additionally identified more FP CNVs. Moreover, the CNV composition differed between the SNP array and the ULP‐WGS‐based CNV callers, with ichorCNA and WisecondorX showing the most similar CNV detection pattern. While SNP arrays remain valuable in certain contexts due to their established use and familiarity in clinical settings, ULP‐WGS‐based CNV callers offer a more robust approach, especially for analyzing FFPE samples. Unlike SNP arrays, which require 40 ng of gDNA input of good quality [39], ULP‐WGS requires only 6 ng of gDNA, making it more suitable for low input FFPE samples (including those from specimens that are 4–5 years old). While SNP arrays tended to generate more false positive results, ULP‐WGS showed greater accuracy in handling such cases. This robustness, combined with its lower DNA input, makes ULP‐WGS a promising alternative for FFPE samples analysis, without necessarily compromising performance.

Our findings underscore the importance of various pre‐analytical parameters, that is, specimen type, age of FFPE samples, gDNA input, and total coverage depth. The detection of CNV events improves significantly as the percentage of neoplastic cells increases, likely because the tumor signal is easier to differentiate from the background noise. Besides that, higher neoplastic cell content ensures a more accurate CN measurement, which is critical for ensuring accurate CNV profiling in heterogeneous tumors. Achieving tumor purity above 50% can be challenging in routine clinical samples, even when using macro‐ or microdissection. We recommend being cautious when interpreting the results of samples with a neoplastic cell content below 50%. Chen *et al*. [40] reported that low tumor purity (< 50%) greatly affected CNV calling by missing most calls (both AMP and DEL), lowering CNs for amplification calls and increasing CNs for deletion calls. Medium purity (50% and 75%) showed a similar trend, but with less intensity. Their reported effects of low input DNA amount on CNV calling were relatively mild. In another study, tumor cell enrichment (by using tissue suspensions) in gastric cancer FFPE samples increased the number of CNVs detected via NGS and contributes to an improvement in CN. On the other hand, this enrichment method was considered unsuitable for CNV detection in cases with high tumor content [41]. tNGS‐based methods face similar limitations regarding low tumor content. SNP array–based approaches, however, exhibit somewhat improved performance under these conditions [42]. Another important factor influencing detection sensitivity is the magnitude of the copy number variation itself; large amplifications are generally easier to detect than subtle deviations near diploid levels. Cross‐platform comparisons of copy number magnitudes should be made cautiously, as some methods (e.g., FISH) yield more precise copy number estimates than coverage‐based approaches such as tNGS, EPIC array or ULP‐WGS. An illustrative example of this discrepancy is provided in Fig. S2A,B, where the same *MET* amplification yields different apparent copy number magnitudes across platforms.

The results of this study highlight the importance of optimizing analytic parameters in a CNV detection protocol to obtain reliable results. The choice of bin size is a trade‐off between sensitivity (smaller bin sizes) and specificity (larger bin sizes), and the optimal bin size may vary depending on the type of CNV being analyzed. Smaller bin sizes generally allow the software to detect smaller CNV events with finer resolution [43]. This, however, tends to introduce more noise, potentially leading to a higher number of FP [29, 30], which is evident from the high number of FP detected with CNVpytor when using a smaller bin size. ichorCNA shows little difference between 500 kb and 1 Mb for FFPE samples but performs poorly with smaller bin sizes. With WisecondorX, bin size reduction from 1 Mb to 250 kb is beneficial, without affecting FP detection. However, a bin size lower than 250 kb is not advisable. WisecondorX detected all TP until the coverage was reduced below 0.1×, whereas CNVpytor and ichorCNA required a coverage depth of at least 0.25×. While lower coverage depths led to a higher MAD score, it did not increase the number of FP, making TP loss the main concern. A key advantage of reducing coverage depth is the ability to process more samples per sequencing run, reducing overall costs.

Analysis of an independent validation set using WisecondorX demonstrated a high detection rate (92%) for CNVs, with the highest detection efficiency observed in DEL. Importantly, even when the detected CNV size is small (< 5 Mbp), high |*Z*‐scores| (> 50) can be achieved, suggesting robust detection is possible for both gene‐level and larger genomic regions. In general, the absCN of the CNV had a large influence on |*Z*‐scores|, with low CN (0–5) showing a wide range of  |*Z*‐scores|, while high CN consistently produced high |*Z*‐scores| (mean 95.7). Overall, the results confirm WisecondorX as a reliable tool for detecting CNVs with varying sizes and copy numbers in FFPE samples.

Among the CNV callers evaluated, ichorCNA and WisecondorX demonstrated comparable performance on FFPE samples. The deviant performance of CNVpytor may be partly due to the omission of allele‐imbalance sub‐analysis, which was not feasible given the limited coverage depth of ULP‐WGS sequencing. Rather than selecting a single CNV caller, an alternative strategy is to apply a majority rule, considering only CNVs detected by both tools (i.e., consensus calls). This conservative approach prioritizes confidence over quantity. Precision can be further enhanced by applying stricter copy number thresholds (e.g., CN > 3 for amplifications and CN < 1 for deletions) which reduce the number of FP while retaining TP calls. When implementing a CNV assay as a supplementary diagnostic tool to confirm cancer diagnosis, high specificity and low false positive rates are essential to minimize overtreatment. In this context, WisecondorX is the most suitable option for CNV detection in FFPE samples. As an open‐source tool, it can be seamlessly integrated into existing bioinformatics pipelines. Combined with ULP‐WGS, it offers a cost‐effective alternative to SNP arrays or tNGS panels, which typically involve higher reagent and infrastructure costs.

Turnaround time (TAT) is another key consideration for clinical implementation. SNP array analysis typically requires several weeks, with 4–5 days of hands‐on processing and batching of samples, which limits its practicality for routine diagnostics. tNGS assays typically return results within 1 or 2 weeks. ULP‐WGS offers a comparable TAT to tNGS and can be performed in parallel on the same sequencing platform. While FISH analysis can deliver results within 1 week, it is limited to single marker assessment. Collectively, these features position ULP‐WGS as a robust, cost‐effective, and readily implementable method for detecting clinically relevant CNVs in FFPE tumor samples.

This study involved an extensive validation using a large number of samples to thoroughly assess the performance of ULP‐WGS‐based CNV callers in comparison to SNP arrays. Beyond evaluating detection accuracy, we also examined the robustness of these methods across diverse sample conditions. However, one of the key challenges in our analysis is the lack of complete knowledge of all true CNVs present in the samples of our training and validation datasets. This limitation makes it difficult to draw definitive conclusions about the rate of false positive CNV detections. We tried to mitigate this lack by also evaluating non‐tumor samples, which should in theory contain zero or one CNV. Another limitation of this study is that DNA quality was not systematically assessed for all samples, and the impact of low‐quality DNA on the results remains to be determined. Additionally, one of the remaining hurdles is the dependency on high neoplastic cell content in FFPE samples for reliable CNV detection. Further efforts are needed to refine these methods and reduce their reliance on high tumor purity, ensuring that CNV detection remains accurate and robust even in samples with lower neoplastic cell content. Addressing this challenge will be important for improving the clinical applicability of ULP‐WGS‐based CNV detection in routine diagnostics.

## 5 Conclusion

With the increasing significance of CNV detection in molecular pathology for accurate diagnoses and predicting responses to targeted therapies in various solid tumors, it becomes crucial to address the challenges of detecting CNVs in DNA derived from FFPE samples. In our study, we assessed the robustness of different CNV callers using ULP‐WGS data from FFPE samples and compared their performance with SNP‐based array analysis. We evaluated various ULP‐WGS‐based CNV callers and analyzed the impact of pre‐analytical parameters—including neoplastic cell content, the age of the FFPE samples, and DNA concentration—as well as analytical parameters such as bin size, on the accuracy of CNV detection. Our findings emphasize the importance of optimization of these parameters. Additionally, our study highlights the crucial role of high tumor content in ensuring reliable CNV analysis in FFPE samples. This research demonstrates the potential for utilizing archived FFPE samples in genomic studies and cancer diagnostics.

## Conflict of interest

The authors declare no conflict of interest.

## Author contributions

HG, KZ, SK, and VS conceptualized the study. HG and KZ performed the experiments and data analysis. VS, AS, and SK provided histopathological review of the samples. ER aided HG with the statistical analysis. SH and KZ set up the ULP‐WGS analysis pipeline. LCK, EB, LS, and KDW provided molecular insights. All authors contributed to the manuscript revision, read, and approved the submitted version.

## Supporting information


**Fig. S1.** Genome‐wide CNV detection profiles of one sample.

**Fig. S2.** Cross‐platform comparison of *MET* amplification magnitudes in a small biopsy with 50% tumor cell content.

**Table S1.** Tumor samples and their respective CNVs used for the training dataset (a), determining the limit of detection (b), and independent validation dataset (c).

**Table S2.** FFPE samples of varying tissue types used to assess the robustness of copy number profiling.

## Data Availability

The data that support the findings of this study are available upon reasonable request from the corresponding author (karen.zwaenepoel@uantwerpen.be). The data are not publicly available due to privacy or ethical restrictions.
