# Supplementary material for: Crucial parameters for precise copy number variation detection in formalin‐fixed paraffin‐embedded solid cancer samples
Source: Mol Oncol. 2025 Dec 23;20(5):1270–83. doi: 10.1002/1878-0261.70192 (PMC13155139; doi:10.1002/1878-0261.70192)
Supplement: Supplementary file 1 — Fig. S1. Genome‐wide CNV detection profiles of one sample. [file MOL2-20-1270-s001.pdf]

A

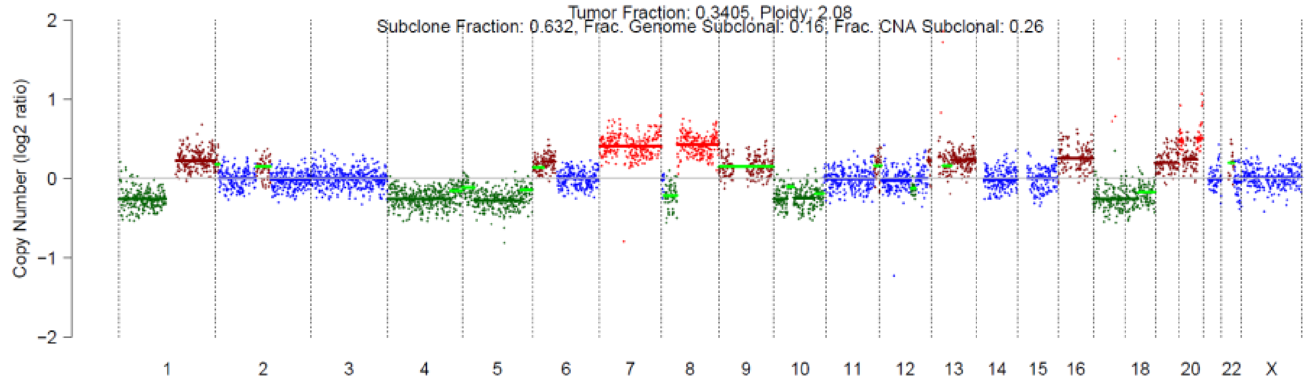

B

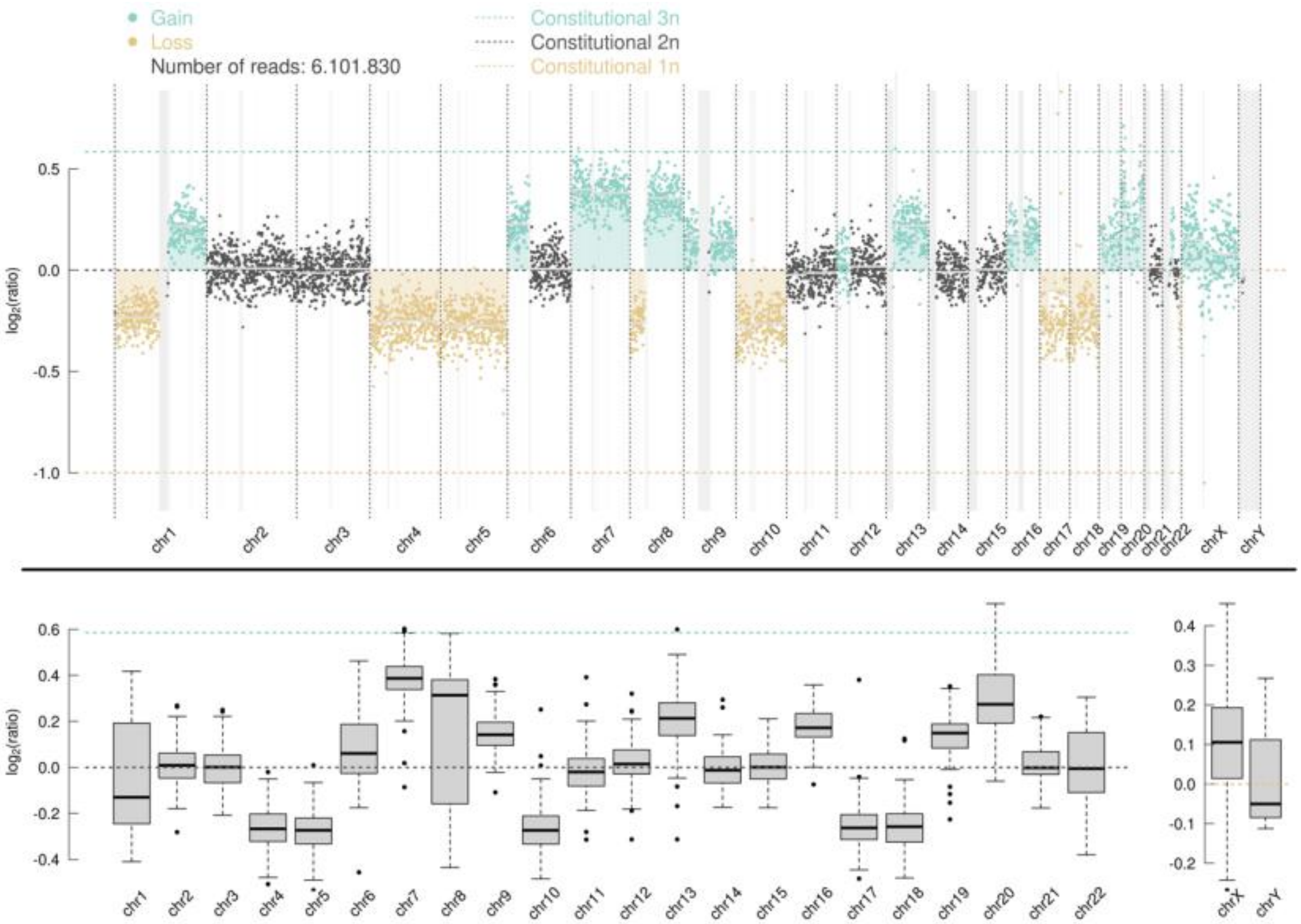

C

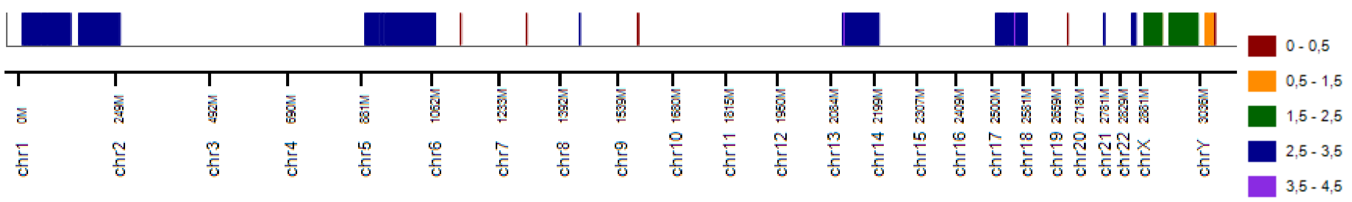

Supplementary figure 1. Genome-wide CNV detection profiles of one sample. (A) CNV detection profile generated by ichorCNA with bin size 500 kb, QC30 mapping and |logR| = 0.2. (B) CNV detection profile generated by WisecondorX with bin size 500 kb and |Z| = 5. The boxplots show the median values, the interquartile range (IQR), and the whiskers extending to the data points within 1.5 × IQR, with outliers plotted as dots. (C) CNV detection profile generated by SNP array with bin size 500 kb, confidence threshold 35 and minimum probe count 3. A CNV detection profile for CNVpytor could not be included as we were not able to run the entire algorithm, since coverage depth did not allow us to perform CNV calling with CNVpytor from B-allele frequency (BAF) data. The X-axis shows each chromosome. The Y-axis represents the $\log_2$ ratio of the detected copy number.
