# Supplementary material for: Crucial parameters for precise copy number variation detection in formalin‐fixed paraffin‐embedded solid cancer samples
Source: Mol Oncol. 2025 Dec 23;20(5):1270–83. doi: 10.1002/1878-0261.70192 (PMC13155139; doi:10.1002/1878-0261.70192)
Supplement: Supplementary file 2 — Fig. S2. Cross‐platform comparison of MET amplification magnitudes in a small biopsy with 50% tumor cell content. [file MOL2-20-1270-s002.pdf]

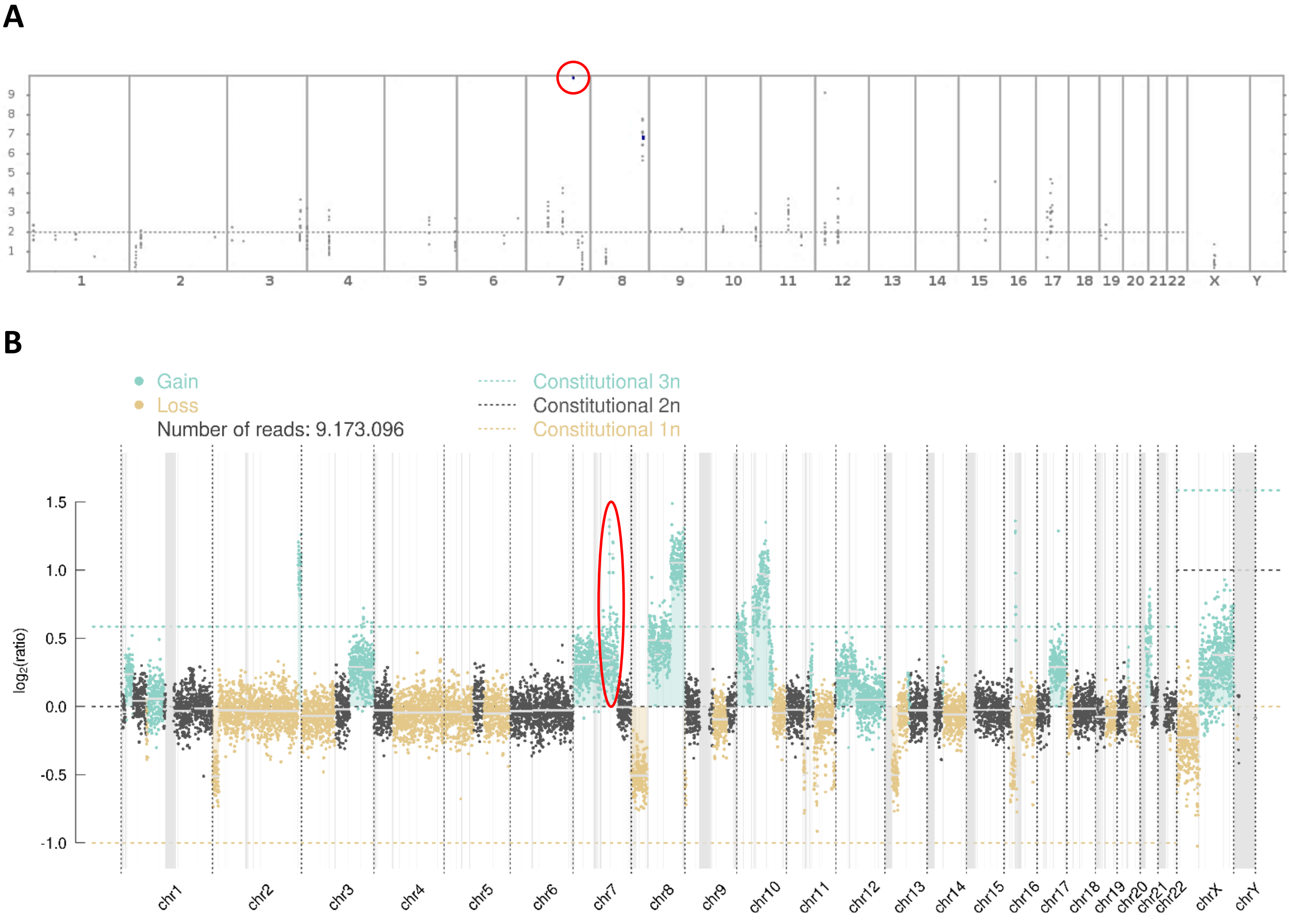


**Supplementary figure 2.** Cross-platform comparison of *MET* amplification magnitudes in a small biopsy with 50% tumor cell content. (A) CNV segment data from routine tNGS result. The X-axis shows each chromosome, the Y-axis represents the detected copy number; the *MET* amplification corresponds to a detected copy number of 10. (B) Genome-wide CNV detection profile generated by WisecondorX, using a 250 kb bin size and |Z-score| = 10. The X-axis shows each chromosome, the Y-axis represents the $log_2$ ratio of the detected copy number; the *MET* amplification corresponds to a detected copy number of 5.3. The *MET* amplification is circled in red in both CNV profiles.
