# Supplementary material for: Crucial parameters for precise copy number variation detection in formalin‐fixed paraffin‐embedded solid cancer samples
Source: Mol Oncol. 2025 Dec 23;20(5):1270–83. doi: 10.1002/1878-0261.70192 (PMC13155139; doi:10.1002/1878-0261.70192)
Supplement: Supplementary file 3 — Table S1. Tumor samples and their respective CNVs used for the training dataset (a), determining the limit of detection (b), and independent validation dataset (c). [file MOL2-20-1270-s003.pdf]

Supplementary table 1. Tumor samples and their respective CNVs used for the training dataset (a), determining the limit of detection (b), and independent validation dataset (c). CN = copy number; dPCR = digital polymerase chain reaction; FISH = fluorescence in situ hybridization; IHC = immunohistochemistry; (t)NGS OFA = (targeted) next-generation sequencing (Oncomine Focus Assay).

Sample	Organ	Tissue type	Dataset	Neoplastic cells (%)	Detection technique	CNV	CN	CNV type	GRCh37 Genomic location	Size (kb)
1	NA	commercial reference material	a	100	dPCR, NGS	*AKT2*	5	LLAMP	Chr 19: 40,736,224 - 40,791,252	55
						BRAF	5	LLAMP	Chr 7: 140,413,128 - 140,624,729	212
						EGFR	5	LLAMP	Chr 7: 55,086,710 - 55,279,321	193
						ERBB2	5	LLAMP	Chr 17: 37,844,347 - 37,884,911	41
						FGFR3	5	LLAMP	Chr 4: 1,795,020 - 1,810,594	16
						KIT	5	LLAMP	Chr 4: 55,524,124 - 55,606,881	83
						KRAS	5	LLAMP	Chr 12: 25,358,180 - 25,403,863	46
						MET	5	LLAMP	Chr 7: 116,312,250 - 116,438,431	126
						MYC	5	LLAMP	Chr 8: 128,747,680 - 128,755,197	8
						MYCN	5	LLAMP	Chr 2: 16,080,672 - 16,087,126	6
						NTRK1	5	LLAMP	Chr 1: 156,785,542 - 156,851,642	66
						PIK3CA	5	LLAMP	Chr 3: 178,866,145 - 178,957,881	92
2	bile duct	resection	a	15	FISH, IHC	*MDM2*	10	LLAMP	Chr 12: 68,808,172 - 68,845,544	37
3	lung	biopsy	a	15	FISH, tNGS OFA - read depth	*MDM2*	10	LLAMP	Chr 12: 68,808,172 - 68,845,544	37
4	brain	resection	a	>80	Infinium MethylationEPIC array	*EGFR*	2,8	LLAMP	Chr 7: 55,086,710 - 55,279,321	193
						MYCN	3,2	LLAMP	Chr 2: 16,080,672 - 16,087,126	6
						CDKN2A	0	DEL	Chr 9: 21,967,752 - 21,995,324	28
						10q loss	1,5	DEL	Chr 10: 40,200,000 - 135,534,747	95335
5	lung	biopsy	a	35	tNGS OFA - read depth	*EGFR*	97	HLAMP	Chr 7: 55,086,710 - 55,279,321	193
6	brain	biopsy	a	50	tNGS glioma - read depth	*EGFR*	109	HLAMP	Chr 7: 55,086,710 - 55,279,321	193
						CDKN2A	0	DEL	Chr 9: 21,967,752 - 21,995,324	28
7	colon	resection	a	40	FISH, tNGS OFA - read depth	*HER2*	169	HLAMP	Chr 17: 39,688,094 - 39,728,658	41
8	lung	biopsy	a	80	tNGS OFA - read depth	*FGFR1*	142	HLAMP	Chr 8: 38,411,143 - 38,468,635	57
9	brain	biopsy	a	40	tNGS glioma - read depth	*CDKN2A*	0	DEL	Chr 9: 21,967,752 - 21,995,324	28
10	brain	biopsy	a	50	NGS SNPitty	*1p loss*	1,5	DEL	Chr 1: 1 - 123,400,000	123400
						19q loss	1,5	DEL	Chr 19: 26,200,001 - 58,617,616	32418
11	brain	resection	b	>70	Infinium MethylationEPIC array	*MET gain*	2,8	LLAMP	Chr 7: 116,312,250 - 116,438,431	126
						1q gain	2,5	LLAMP	Chr 1: 123,514,337 - 248,956,422	125442
						2q gain	2,5	LLAMP	Chr 2: 93,300,001 - 243,199,373	243199
						7q gain	2,5	LLAMP	Chr 7: 59,900,001 - 159,138,663	159138
						20 gain	2,5	LLAMP	Chr 20: 1 - 63,025,520	63025
						1p loss	1,4	DEL	Chr 1: 1 - 123,400,000	123400
						3p loss	1,4	DEL	Chr 3: 1 - 91,000,000	91000
						4 loss	1,4	DEL	Chr 4: 1 - 191,154,276	191154
						6q loss	1,5	DEL	Chr 6: 61,000,001 - 171,115,067	110115
						10 loss	1,4	DEL	Chr 10: 1 - 135,534,747	135534
						18 loss	1,4	DEL	Chr 18: 1 - 78,077,248	78077
						22 loss	1,4	DEL	Chr 22: 1 - 51,304,566	51304
12	brain	biopsy	b	70	Infinium MethylationEPIC array, IHC	*CDKN2A*	0	DEL	Chr 9: 21,967,752 - 21,995,324	28
13	brain	resection	c	40	tNGS glioma - read depth	*EGFR*	28	HLAMP	Chr 7: 55,086,710 - 55,279,321	193
14	lung	cytology	c	35	FISH, tNGS OFA - read depth	*MET*	16	HLAMP	Chr 7: 116,312,250 - 116,438,431	126
15	lung	biopsy	c	40	FISH, tNGS OFA - read depth	*MET*	8	LLAMP	Chr 7: 116,312,250 - 116,438,431	126
16	lung	biopsy	c	50	FISH, tNGS OFA - read depth	*MET*	10	LLAMP	Chr 7: 116,312,250 - 116,438,431	126
17	brain	resection	c	>80	Infinium MethylationEPIC array	*4p gain*	2,6	LLAMP	Chr 4: 1 - 49,989,692	49990
						20q gain	3	LLAMP	Chr 20: 28,162,709 - 64,444,167	36281
18	brain	resection	c	>70	Infinium MethylationEPIC array	*7 gain*	2,5	LLAMP	Chr 7: 1 - 159,138,663	159138
						9p loss	1,6	DEL	Chr 9: 1 - 43,026,791	43027
						20 gain	2,3	LLAMP	Chr 20: 1 - 63,025,520	63025
						22 loss	1,6	DEL	Chr 22: 1 - 51,304,566	51304
						CDKN2A	1,3	DEL	Chr 9: 21,967,752 - 21,995,324	28
19	brain	resection	c	>80	Infinium MethylationEPIC array	*22q loss*	1,6	DEL	Chr 22: 15,029,036 - 50,818,468	35789
20	brain	resection	c	50-70	Infinium MethylationEPIC array	*1q gain*	2,8	LLAMP	Chr 1: 1 - 123,400,000	123400
						7 gain	2,6	LLAMP	Chr 7: 1 - 159,138,663	159138
						10 loss	2,7	DEL	Chr 10: 1 - 135,534,747	135534
						19 gain	2,5	LLAMP	Chr 19: 1 - 59,128,983	59128
						20q gain	2,6	LLAMP	Chr 20: 28,162,709 - 64,444,167	36281
21	brain	resection	c	>80	Infinium MethylationEPIC array	*1p gain*	2,4	LLAMP	Chr 1: 1 - 123,400,000	123400
						7 gain	2,3	LLAMP	Chr 7: 1 - 159,138,663	159138
						10p loss	1,6	DEL	Chr 10: 1 - 39,826,524	39827
						14q loss	1,8	DEL	Chr 14: 17,217,953 - 107,043,718	99826
						20q gain	2,3	LLAMP	Chr 20: 28,162,709 - 64,444,167	36281
						22q loss	1,5	DEL	Chr 22: 15,029,036 - 50,818,468	35789
22	brain	resection	c	>80	Infinium MethylationEPIC array	*1p loss*	1,5	DEL	Chr 1: 1 - 123,400,000	123400
						19q loss	1,5	DEL	Chr 19: 26,200,001 - 58,617,616	32418
23	brain	resection	c	>70	Infinium MethylationEPIC array	*1p gain*	2,5	LLAMP	Chr 1: 1 - 123,400,000	123400
						7 gain	2,5	LLAMP	Chr 7: 1 - 159,138,663	159138
						3 loss	1,6	DEL	Chr 3: 1 - 198,022,430	198022
						9q loss	1,7	DEL	Chr 9: 43,138,606 - 138,394,717	95256
24	breast	biopsy	c	20	FISH	*HER2/ERBB2*	20	HLAMP	Chr 17: 37,844,347 - 37,884,911	41
25	breast	biopsy	c	30	FISH	*HER2/ERBB2*	12	LLAMP	Chr 17: 37,844,347 - 37,884,911	41
26	breast	biopsy	c	50	FISH, IHC	*HER2/ERBB2*	20	HLAMP	Chr 17: 37,844,347 - 37,884,911	41
27	breast	biopsy	c	50	FISH, IHC	*HER2/ERBB2*	12	LLAMP	Chr 17: 37,844,347 - 37,884,911	41
28	skin	biopsy	c	80-90	IHC	*BAP1*	1	DEL	Chr 3: 52,435,024 - 52,444,024	9
29	fat	biopsy	c	70	FISH	*MDM2*	20	HLAMP	Chr 12: 68,808,172 - 68,845,544	37

30	lung	biopsy	c	>50	FISH, tNGS OFA - read depth	*MET*	25	HLAMP	chr 7: 116,312,250 - 116,438,431	1
31	lung	resection	c	40	FISH, tNGS OFA - read depth	*CDK4*	11,1	HLAMP	chr 12: 57,747,727 - 57,756,013	1
32	oesophagus	biopsy	c	30	FISH	*HER2*	30	HLAMP	chr 17: 39,688,094 - 39,728,658	56
