# Supplementary material for: Crucial parameters for precise copy number variation detection in formalin‐fixed paraffin‐embedded solid cancer samples
Source: Mol Oncol. 2025 Dec 23;20(5):1270–83. doi: 10.1002/1878-0261.70192 (PMC13155139; doi:10.1002/1878-0261.70192)
Supplement: Supplementary file 4 — Table S2. FFPE samples of varying tissue types used to assess the robustness of copy number profiling. [file MOL2-20-1270-s004.pdf]

Supplementary table 2. FFPE samples of varying tissue types used to assess the robustness of copy number profiling.

Sample	Sample type	Age of sample (years)	Neoplastic cells (%)
1	Biopsy	5	40-50
2	Biopsy	4	70-80
3	Biopsy	4	80-90
4	Biopsy	4	10
5	Biopsy	4	5-20
6	Biopsy	5	30-50
7	Biopsy	3	50
8	Biopsy	3	10-30
9	Biopsy	4	30-40
10	Biopsy	4	50-60
11	Biopsy	3	30
12	Resection	4	15
13	Resection	3	>50
14	Resection	3	50
15	Resection	3	>50
16	Resection	3	35
17	Resection	4	50
18	Resection	4	>50
19	Biopsy	2	50-60
20	Biopsy	2	80
21	Biopsy	2	40-50
22	Biopsy	2	60-80
23	Biopsy	2	50-70
24	Biopsy	2	30-50
25	Biopsy	3	20-30
26	Biopsy	2	30
27	Biopsy	0	60
28	Biopsy	2	10-20
29	Biopsy	2	10-30
30	Biopsy	2	50-60
31	Biopsy	2	50
32	Biopsy	2	10
33	Biopsy	2	80
34	Biopsy	3	50-60
35	Biopsy	2	50
36	Biopsy	2	40-60
37	Biopsy	3	40-50
38	Biopsy	0	>50
39	Biopsy	2	10-15
40	Biopsy	2	15
41	Biopsy	2	60-75
42	Biopsy	2	25-50
43	Biopsy	3	80
44	Biopsy	3	60-80
45	Biopsy	3	30-40
46	Biopsy	3	40-60
47	Biopsy	3	20-30
48	Biopsy	3	40-50

49	Biopsy	2	30
50	Biopsy	3	20-50
51	Biopsy	3	20-30
52	Biopsy	0	70
53	Biopsy	3	70-80
54	Biopsy	3	70-80
55	Biopsy	3	10-30
56	Biopsy	3	10-20
57	Biopsy	2	10
58	Biopsy	2	10-15
59	Biopsy	3	10-15
60	Biopsy	3	20-30
61	Biopsy	2	10-15
62	Biopsy	2	30-40
63	Biopsy	2	25-35
64	Biopsy	2	50-60
65	Biopsy	0	35
66	Biopsy	0	20
67	Biopsy	0	50
68	Resection	2	50
69	Resection	0	15
70	Resection	0	>50
71	Resection	0	>50
72	Resection	0	70
73	Resection	0	35
74	Biopsy	1	50
75	Biopsy	1	40-50
76	Biopsy	1	40-60
77	Biopsy	1	30-50
78	Biopsy	1	40-50
79	Biopsy	1	10
80	Biopsy	2	30-50
81	Biopsy	2	15-35
82	Biopsy	2	10-20
83	Biopsy	2	40-50
84	Biopsy	2	80-90
85	Biopsy	2	5-10
86	Biopsy	2	10-20
87	Biopsy	0	50
88	Biopsy	0	50
89	Biopsy	0	50
90	Biopsy	2	70-80
91	Biopsy	1	50-70
92	Biopsy	0	35
93	Biopsy	2	<10
94	Biopsy	0	35
95	Biopsy	2	20-30
96	Biopsy	2	20-30
97	Biopsy	1	25-35
98	Biopsy	0	35
99	Biopsy	0	50

100	Biopsy	0	50
101	Biopsy	0	10
102	Biopsy	0	50
103	Resection	0	>50
104	Resection	0	70
105	Biopsy	0	15
106	Biopsy	1	50-60
107	Biopsy	1	80
108	Biopsy	1	30-50
109	Biopsy	1	50-70
110	Biopsy	1	30-50
111	Biopsy	0	>50
112	Resection	0	50
113	Biopsy	0	50
114	Biopsy	0	50
115	Biopsy	0	50
116	Biopsy	0	75
117	Biopsy	0	50
118	Biopsy	0	40
119	Biopsy	0	50
120	Biopsy	0	50
121	Resection	0	15
122	Biopsy	0	35
123	Biopsy	0	15
124	Biopsy	0	80
125	Resection	0	40
126	Biopsy	0	>80
127	Biopsy	8	12
128	Biopsy	0	20
